# Identification and validation of a T cell marker gene-based signature to predict prognosis and immunotherapy response in gastric cancer

**DOI:** 10.1038/s41598-023-48930-8

**Published:** 2023-12-04

**Authors:** Jinlin Zhong, Rongling Pan, Miao Gao, Yuqian Mo, Xin Peng, Guoxiao Liang, Zixuan Chen, Jinlin Du, Zhigang Huang

**Affiliations:** 1 Department of Epidemiology and Health Statistics, School of Public Health, Guangdong Medical University, Dongguan, Guangdong People’s Republic of China; 2 Key Laboratory of Noncommunicable Diseases Control and Health Data Statistics of Guangdong Medical University, Dongguan, Guangdong People’s Republic of China

**Keywords:** Cancer, Computational biology and bioinformatics, Biomarkers, Oncology

## Abstract

Although the role of T cells in tumor immunity and modulation of the tumor microenvironment (TME) has been extensively studied, their precise involvement in gastric adenocarcinoma remains inadequately explored. In this work, we analyzed the single-cell RNA sequencing data set in GSE183904 and identified 322 T cell marker genes using the “FindAllMarkers” method of the R package “Seurat”. STAD patients in the TCGA database were divided into high-risk and low-risk categories based on risk scores. The five-gene prediction signature based on T cell marker genes can predict the prognosis of gastric cancer patients with high accuracy. In the training cohort, the areas under the receiver operating characteristic (ROC) curve were 0.667, 0.73, and 0.818 at 1, 3, and 5 years. External validation of the predictive signature was also performed using multiple clinical subgroups and GEO cohorts. To help with practical application, a diagnostic model was created that shows values of 0.732, 0.752, and 0.816 for the relevant areas under the ROC curve at 1, 3, and 5 years. The T cell marker genes identified in this study may serve as potential therapeutic targets, and the developed predictive signatures and nomograms may aid in the clinical management of gastric cancer.

## Introduction

Being the 3rd greatest cause of worldwide cancer mortality and the 5th most prevalent cancer, gastric cancer is an urgent issue for the public^1^. The most frequently diagnosed histological subtype of gastric cancer is stomach adenocarcinoma (STAD). Currently, a variety of therapeutic options including endoscopic therapy, surgical therapy, radiotherapy, and chemotherapy are used to manage this disease^2^. Several biomarkers associated with gastric cancer, such as HER2, MSI, PD-L1, VEGF, and VEGFR-2^3,4^ have been identified for screening, diagnosis, typing, targeted therapy, and monitoring of the disease. However, no single biomarker can comprehensively address all aspects of gastric cancer. As a result, there is a strong need to find more reliable biomarkers and develop sensible combination therapies for the best management of stomach cancer.

During tumor development, tumor cells interact with a variety of cells and tissues in their surroundings, including blood vessels, fibroblasts, and lymphocytes. The tumor microenvironment refers to this network of cells as well as the tumor as a whole. Among these cell types, immune cells make up the tumor immune microenvironment^5,6^. Through a variety of processes, the TME contributes significantly to the growth of tumors, and changes in TME can act as biomarkers for immunotherapy. Understanding the TME's function is crucial for creating new cancer immunotherapies. The primary focus of immunotherapy for gastric cancer is to activate T cells and achieve therapeutic effects by targeting the PD-1 molecule^7^. Known also as T cells, T lymphocytes are essential elements of the body's defensive system against malignancies, the tumor microenvironment's immunosuppressive and costimulatory signals regulate how they work.

The characterization of heterogeneous and phenotypically varied cell groups within malignancies has been made feasible by single-cell RNA sequencing (scRNA-seq)^8^. The complexity of the TME and the relationships between cells are shown by scRNA-seq, which differs from traditional “bulk” RNA-sequencing and may present new prospects for discovering new cancer treatment targets. Using scRNA-seq data from GC samples, this study clarified the flag genes of tumor-infiltrating T lymphocytes and their molecular features. Then, we developed a T cell marker gene signature (TCMGS) and evaluated its prognostic potential for STAD using bulk RNA-seq data. We looked into the relationship between the TCMGS's prediction power and the immune therapy response in 3 separate cohorts from the GEO collection.

## Materials and methods

### Data source

The GEO database was used to collect 26 Primary Gastric Tumor Samples from the scRNA-seq data for GSE183904. 410 patients with cancer and 36 healthy people are included in the TCGA database, which was examined for bulk RNA-sequencing data and clinical statistics for GC patients. After tumor patients without any information on survival were disregarded, 383 patients were included in the analysis. GSE62254 (n = 300), GSE84437 (n = 434), and GSE84433 (n = 373) were also chosen to test the model's predictive aptitude using the GEO dataset.

### Method

#### Analysis of scRNA-seq data

scRNA-seq data was processed using the R packages “Seurat” and “SingleR”^9,10^. The scRNA-seq data quality control method started by excluding clusters with less than three cell counts, cells with less than 200 genes that have been mapped, and cells with more than 10% mitochondrial genes. The data was normalized using the “NormalizeData” function. Top 15 principal components (PCs) from the top 2,000 highly variable genes were found using principal component analysis (PCA). Batch effects were solved by utilizing the “Harmony” package. Unsupervised cell placement using T-distributed stochastic neighbor embedding (t-SNE) allowed for the objective depiction of cell subpopulations on a two-dimensional map^11^. Using the “FindAllMarkers” method, we investigated how the gene expression of a cluster varied from that of every other cluster. We utilized the criteria |log (fold change)|> 1 & an adjusted *P*-value < 0.01 to identify marker genes for each cluster. Finally, utilizing reference information from the Human Primary Cell Atlas, the “SingleR” tool was utilized to annotate cell subpopulations of various clusters^12^.

#### Construction and verification of a prognostic model

To assess T-cell marker genes' predictive significance for overall survival in TCGA-STAD patients, we conducted a single-variable Cox regression analysis and identified prognostic genes with a *P*-value < 0.05. To reduce overfitting and choose the best prognostic genes, we used the LASSO approach with Cox proportional hazards regression. This approach is widely used for regression analysis with high-dimensional variables. We employed tenfold cross-validation with the “cv.glmnet” function to determine the optimal model, with the tuning parameter determined by the standard error (1-SE). The predictive gene signatures were identified via a list of non-zero beta coefficients. We conducted a stepwise multivariate Cox regression analysis, integrating prognostic genes' relative risk coefficients and mRNA expression to develop a risk model, to further ascertain the prognostic significance of these gene signatures. The risk score model was generated as follows:

$$TCMG{-}score=\sum i\,{{{Coefficient}}\left(mRN{A}_{i}\right)}\times Expression(mRN{A}_{i})$$

Based on the median cut-off value, patients were separated into low and high-risk categories. We confirmed the prognostic efficacy of the T-cell marker gene signature using the AUC computed by the “survivalROC” program. In the Kaplan–Meier survival analysis, the log-rank test from the “survminer” R package was used to examine the statistical significance of differences. Finally, we used survival analysis and AUC to confirm the signature's ability to predict outcomes in three different GEO datasets.

#### GSVA, ssGSEA, and hallmark pathway enrichment analysis

To identify significant enriched pathways and biological and molecular operating processes of the prioritized gene list, we performed hallmark pathway enrichment analysis with a *P*-value of 0.05 using clusterProfiler. Furthermore, we conducted gene set enrichment analyses utilizing the GSVA (Gene Set Variation Analysis) program with the H dataset sourced from MsigDB, thereby generating scores to assess pathway activity levels in each cell.

#### The development and validation of a nomogram scoring system

We used “rms” software to create prediction nomograms based on independent prognostic evaluations of the full cohort and merged validation set using clinical characteristics and immune-related risk scores. The total score for a particular sample was calculated by adding the scores assigned to each variable in the nomogram scoring technique^13^. Nomogram calibration curves were used to describe how estimated 1,3,5 year survival events related to actual observations.

#### Identification of immune characteristics for the TCMG-score and assessment of immunotherapy

We performed ssGSEA (single sample gene set enrichment analysis) on the TCGA-STAD cohort to calculate immune infiltration scores for 28 immune cell types to determine the relative abundance of immune cell infiltrates in the TME using enrichment scores. Subsequently, we used the Wilcoxon test to assess the statistical significance of the variance in risk scores and immune infiltrates among the five specific genes. The CIBERSORT method was used to assess the proportion of immune cell infiltration in high- and low-risk groups by examining the gene expression patterns of 22 immune cell types^14^. Immune and stromal scores for each group were computed using the ESTIMATE method^15^. The expression levels of immune checkpoint molecules, such as CD247, play a crucial role in predicting patient response to immunotherapy. In light of this, we conducted a meticulous examination of the expression levels of classical immune checkpoint molecules within the high- and low-risk groups. Immune evasion and treatment were evaluated by delivering tumor immune dysfunction and exclusion (TIDE) to low and high-risk groups^7^. TIDE was used to derive TIDE scores, dysfunction scores, and immunological exclusion scores.

#### Mutation analysis

In order to examine the mutation status in low and high-risk groups, we first measured the number of mutations present in each gene within our sample set using somatic mutation data from The Cancer Genome Atlas (TCGA). The “maftools” package was used to create waterfall graphs, and Tumor Mutational Burden (TMB) data were presented.

#### Statistical analysis

Wilcoxon t-test was used to compare categorical variables among various risk categories. To examine the predictive significance of TCMGS and other clinical and pathological traits, both a single-variable and multiple-variable Cox regression analysis were performed. The cutoff for significance was chosen at *P* = 0.05. The “p.adjust” function in R was used to change the *P*-value for multiple testing using the Benjamini–Hochberg method. Data analysis and figure development were done using R software 4.2.2.

#### Ethics approval and consent to participate

Statement that the raw data used in this study does not require any administrative privileges and all data has been anonymized before acquisition (no information about any human is involved). This study statement confirms that all methods were carried out in accordance with relevant guidelines and regulations in the declaration.

## Result

### scRNA-seq data set quality control

**Figure 1:**
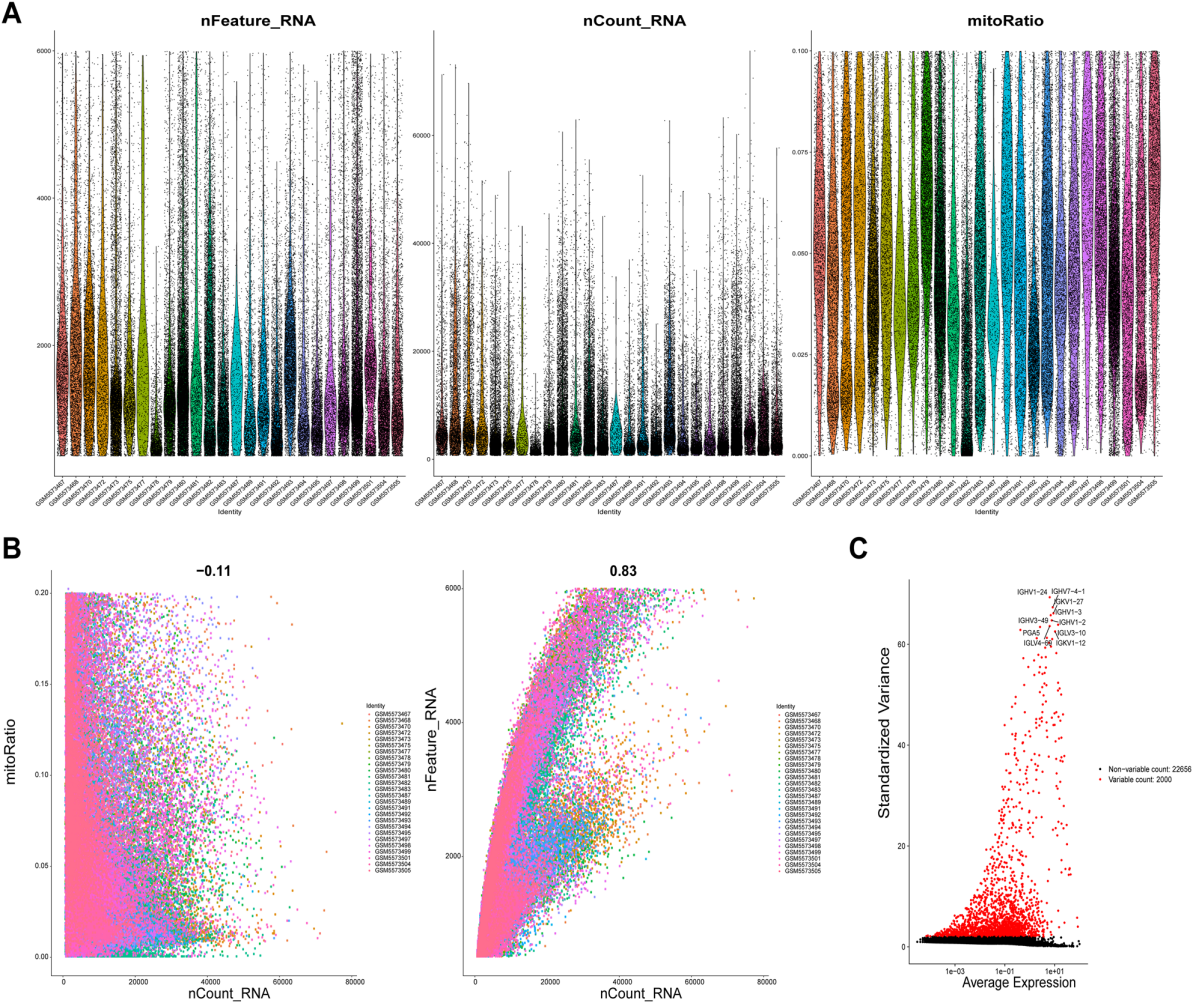
Examination of the GSE183904 data set for flaws: (**A**) displays how the 26 groups of the original GC samples' scRNA-seq data were distributed; relationships between sequencing depth and the mitochondrial genome and total intracellular sequences are shown in (**B**); and the top 10 genes are emphasized in (**C**).

We identified the gene expression patterns of 119,878 cells from 26 GC samples using scRNA-seq data from GSE183904. Figure 1A displays the range of observed gene counts, sequencing depth, and the percentage of mitochondrial content for each sample. According to Fig. 1B, there is a −0.11 association between sequencing depth and mitochondrial gene sequences. The strong positive connection (r = 0.83) between the total number of intracellular sequences and the depth of sequencing suggests that the current RNAs are nuclear transcripts. Figure 1C displays the top 10 genes for expression.

### Profiles of T-cell marker gene expression are identified

**Figure 2:**
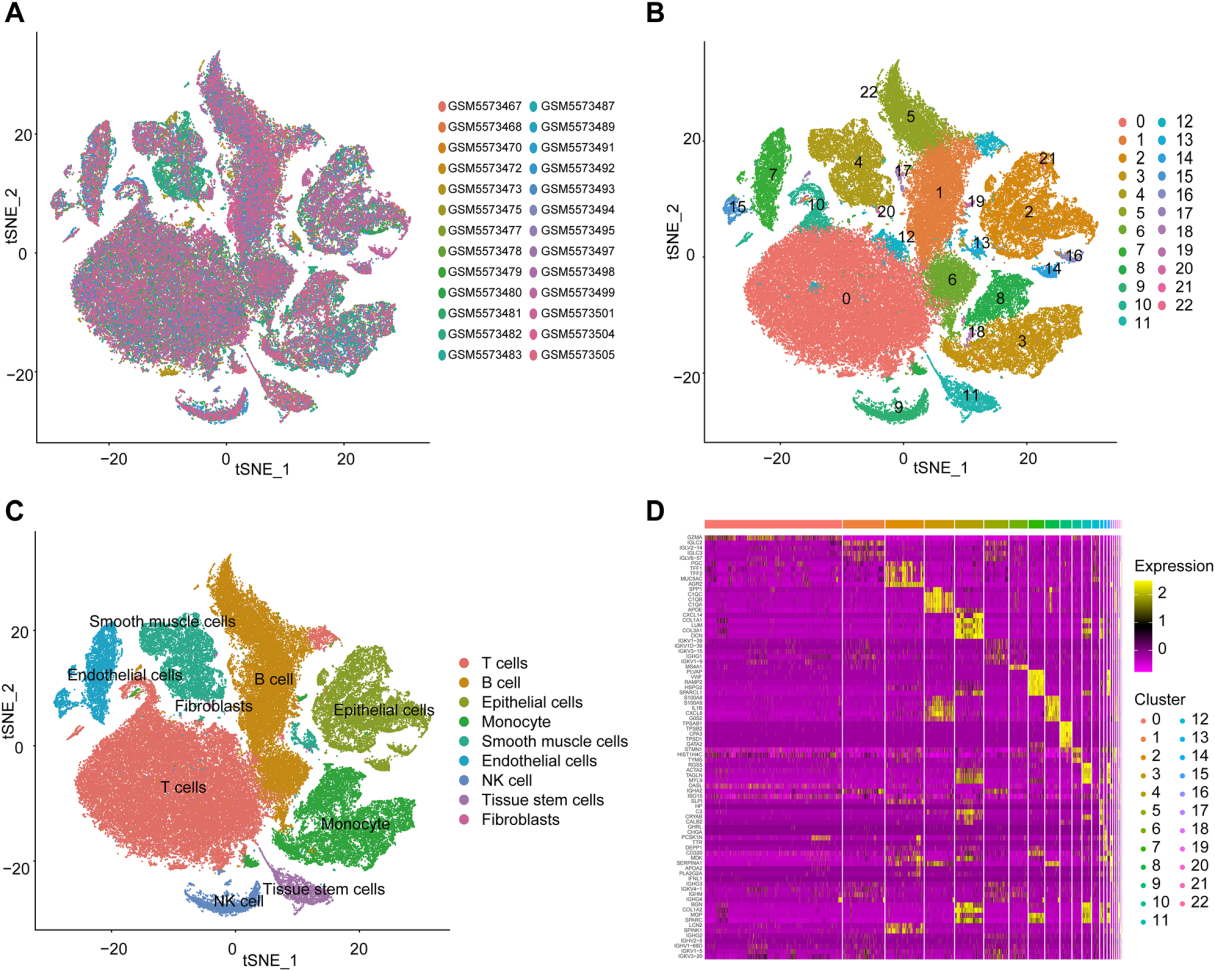
Analysis of the cell types identified by the marker genes using single-cell RNA-sequencing; (**A**) 119,878 cells from 26 GC samples are shown in an t-SNE plot; (**B**) Different cell clusters; (**C**) Cell types identified by marker genes; (**D**) The top five marker genes in each cell cluster are displayed as a heatmap.

Following rigorous quality control screening to remove cells of poor quality, 98,203 cells were used in the ensuing studies (Fig. 2A). The legend in Fig. 2A corresponds to the GSM ID of each sample. PCA was utilized to reduce dimensionality and identify 23 cell clusters using the top 2,000 variable genes (Fig. 2B). Cells in clusters 0, 10, and 12 were identified as T cells using an annotation process that used the Human Primary Cell Atlas reference dataset (Fig. 2C). A heatmap (Fig. 2D) displays the varying levels of marker gene expression within each cluster. Finally, based on |logFC|> 1 & a fatal adjusted *P*-value of 0.05, we identified 322 T-cell marker genes for GC (Table S1).

### Constructing and evaluating a prognostic model

**Figure 3:**
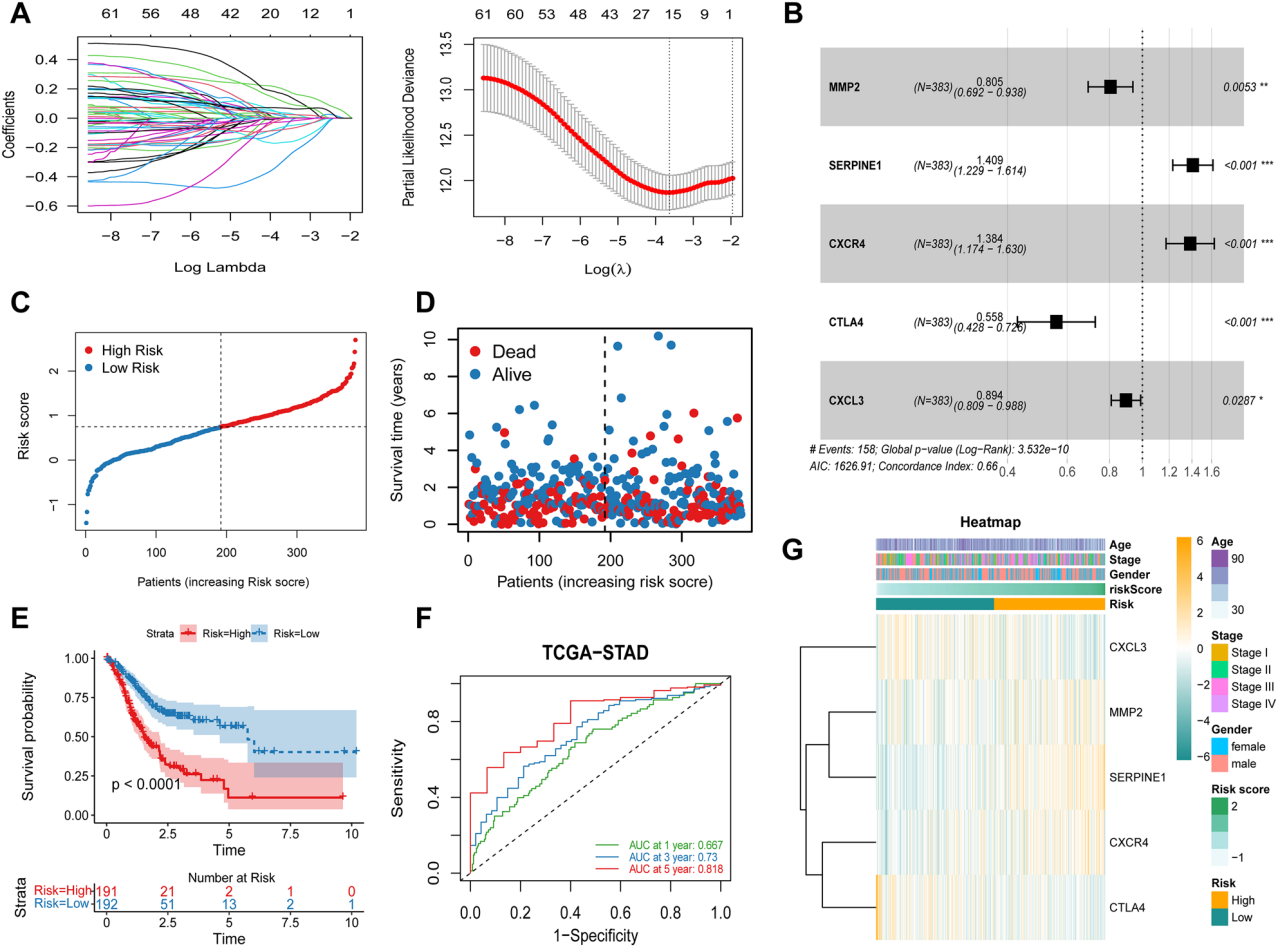
Building and testing prognostic models includes. (**A**) LASSO regression of OS-related genes; (**B**) Forest plot illustrating the outcomes of the multivariate Cox regression; (**C**, **D**) The distribution of risk score and survival status; (**E**) The cohort's Kaplan–Meier curves from the TCGA; (**F**) TCGA-STAD cohort's prognostic models' AUC at 1, 3, and 5 years; (**G**) Distribution of clinicopathological features between high-risk and low-risk shown in heatmap. ***P < 0.001, **P < 0.01, *P < 0.05.

The TCGA-STAD cohort served as the training set for the univariate Cox regression analysis. We harnessed these 322 T cell marker genes to construct a predictive signature, and within this set, 61 genes exhibited a compelling correlation with overall survival (Fig. S1). On the basis of the ideal lambda value and associated coefficients, LASSO analysis then revealed 15 genes (Fig. 3A). The predictive model known as “TCMG-score” was built using five genes (MMP2, SERPINE1, CXCR4, CTLA4, and CXCL3) as a result of multivariate Cox regression analysis (Fig. 3B). This is how the TCMG-score was determined: Risk score is equal to (−0.217 MMP2 expression) + (0.343 SERPINE1 expression) + (0.325 CXCR4 expression) + (−0.584 CTLA4 expression) + (−0.112 CXCL3 expression). According to the risk score ranking from low to high based on the median risk score of 0.749, patients were divided into low-risk (n = 192) and high-risk (n = 191) groups (Fig. 3C). The distribution of survival status is shown in Fig. 3D. High-risk patients had considerably worse overall survival than low-risk patients, according to a Kaplan–Meier survival study (Fig. 3E). Using time-dependent ROC curves, we evaluated how well the signature predicts OS at 1, 3, 5 years. The corresponding AUC values for these intervals were 0.667, 0.73, and 0.818 (Fig. 3F). The risk model heatmap and clinical features are shown in Fig. 3G.

### Validation of the TCMG-score's predictive value in several cohorts

**Figure 4:**
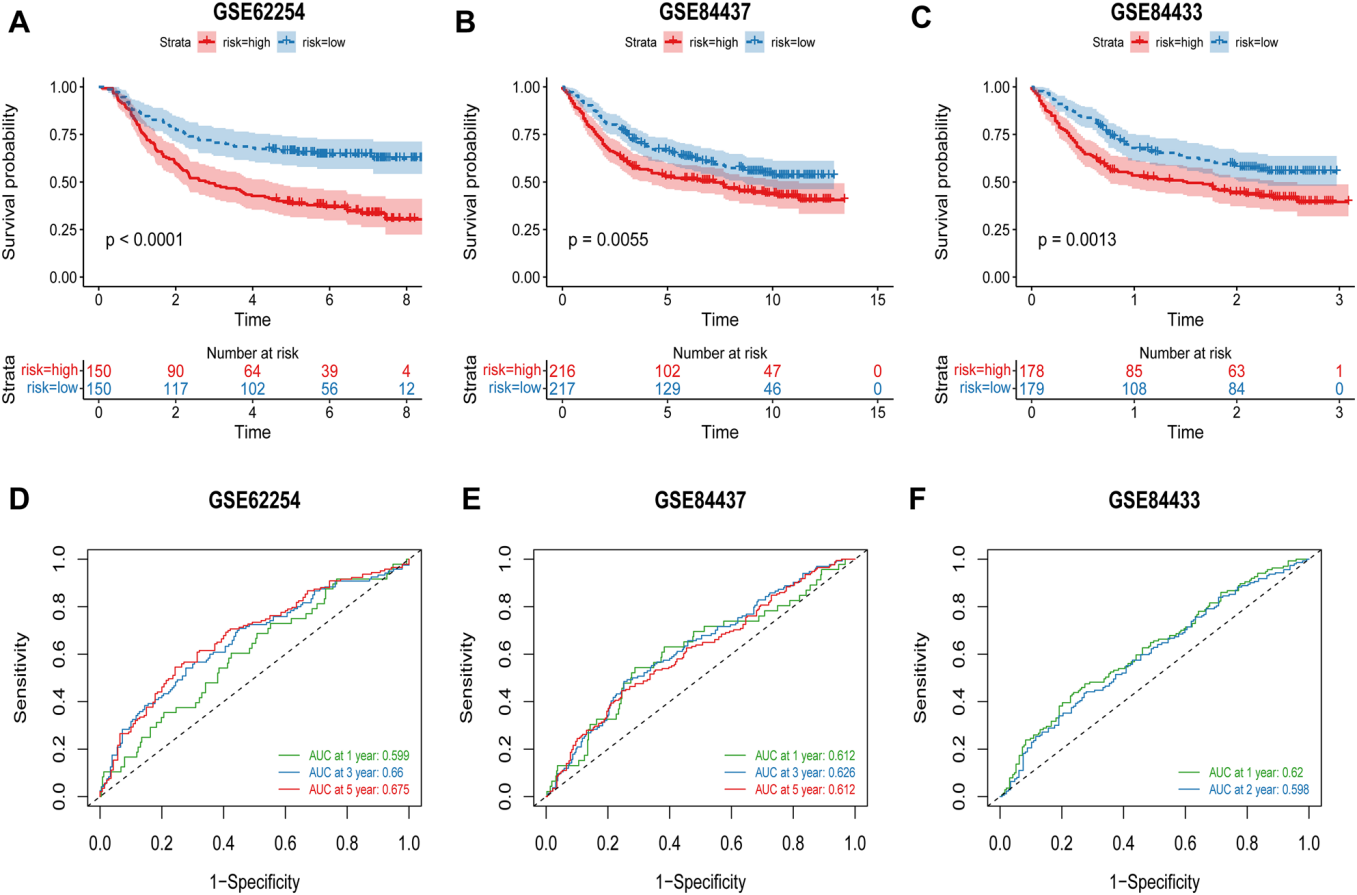
Prognostic model creation and validation. (**A–C**) Every cohort's Kaplan–Meier curves. (**D–F**) The prognostic models' AUC at 1, 3, and 5 years for each cohort.

We evaluated the TCMG-score's performance in three separate GEO cohorts to confirm its prognostic predictive potential. The risk score for each patient in the three cohorts was determined using the same methodology. Based on the median risk score, the patients were separated into low and high-risk subgroups. High-risk patients in both cohorts had a considerably worse prognosis than low-risk ones (Fig. 4A–C). The risk score's ROC curves in the three validation cohorts likewise shown strong performance (Fig. 4D–F).

### Differential expression analysis of TCMG-score subgroups

**Figure 5:**
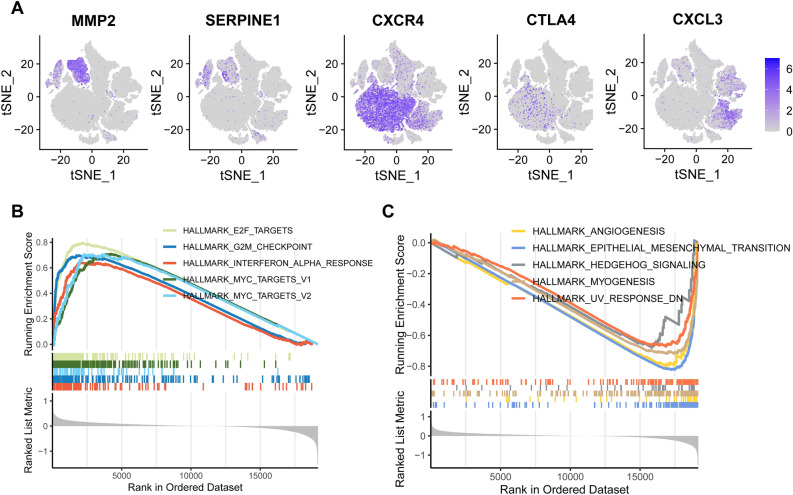
Investigation of the TCMG gene set enrichment. The expression levels of TCMG are depicted in (**A**) using an t-SNE plot. (**B**) Top 5 upregulation pathways for genes enriched in risk scores. (**C**) The top 5 pathways for gene enrichment associated with risk scores that are downregulated.

The 5 core prognostic genes' expression levels are displayed in Fig. 5A. The molecular signature database (MSigDB)'s hallmark was utilized to determine the top 10 key signaling pathways linked to those core genes in GC using GSEA. This allowed us to better understand the functional variations within TCMG-score subgroups and reveal potentially unique protein signatures. When comparing with the low-risk group, E2F is the top hallmark pathway that has increased, whereas epithelial-mesenchymal-transition (EMT) is the top hallmark pathway that has decreased (Fig. 5B, C). As a result, the TCMG has a high association with cancer and may contribute to the genesis and growth of GC.

### TCMGS is related to immune cell infiltration in the TME

**Figure 6:**
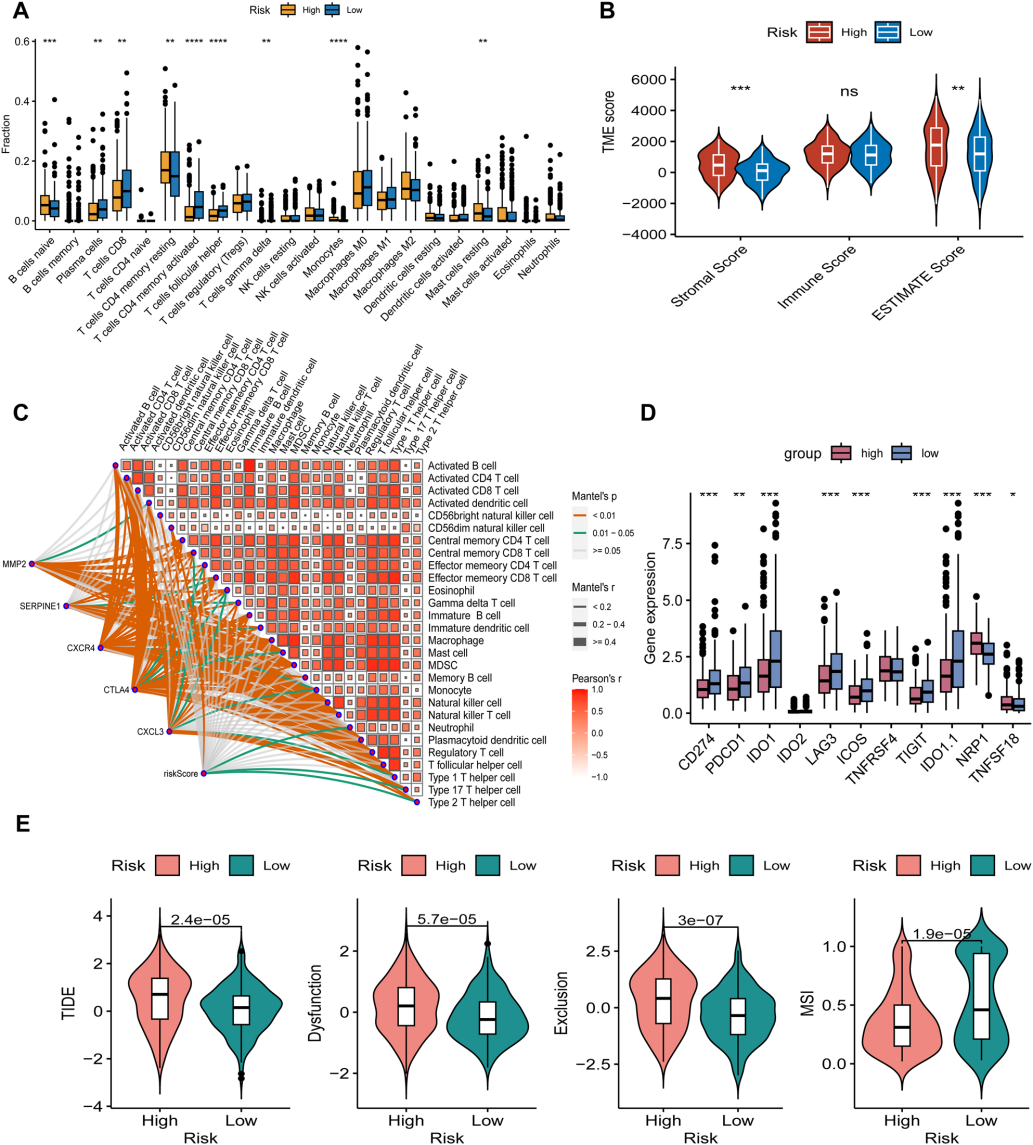
Immune Infiltration in two subgroups of the CAFS score (TCGA). The TCGA cohort's high-risk and low-risk groups' differences in 22 immune cell infiltration are displayed in (**A**)'s boxplot. (**B**) Variations in immunological and stromal scores between high- and low-risk groups. (**C**) Correlation analysis between immune cells and the TCMG-score and 5 potential genes. Immune checkpoint variations across groups at high- and low-risk (**D**). (**E**) Variations in TIDE scores between groups at high- and low-risk.

In the TCGA-STAD cohort, we examined the infiltration status of 26 immune cell types using ssGSEA. Our results showed that the high-risk subgroup had significantly higher proportions of Naive B cells, resting memory CD4 T cells, monocytes, and resting mast cells, whereas the low-risk subgroup had higher frequencies of plasma cells, activated memory CD4 T cells, CD8 T cells and follicular helper T cells (Fig. 6A).

Based on the importance of T cells in the immune response against tumors, we investigated the correlation between TCMG score and immune cell infiltration in gastric cancer patients. Our results showed that patients in the high-risk subgroup had significantly higher immune scores and stromal scores than those in the low-risk subgroup, as determined by the ESTIMATE method (Fig. 6B). This suggests a weak association between the risk score and the level of immune cell infiltration.

The risk score and the five potential genes had a close association with immune cells, as seen in Fig. 6C. We evaluated the expression levels of eight frequently expressed immune checkpoint-related genes in high-risk and low-risk groups in light of the function immune checkpoint inhibitors (ICIs) play in immunotherapy. The low-risk group had strongly expressed levels of PD-L1, PDCD-1, IDO1, LAG-3, ICOS, and TIGIT, according to the findings (Fig. 6D). This study's goal was to assess the potential effectiveness of immunotherapy in various risk groupings in a clinical setting. Given that immune evasion was more likely the higher the TIDE prediction score, patients with high scores were probably not going to benefit from immunotherapy. Low-risk patients were more likely to benefit from ICIs therapy than high-risk patients because they had lower TIDE scores than the high-risk category (Fig. 6E). We also identified differences in T-cell dysfunction, exclusion scores, and microsatellite instability (MSI) scores between the two risk groups.

### Gene mutation analysis

**Figure 7:**
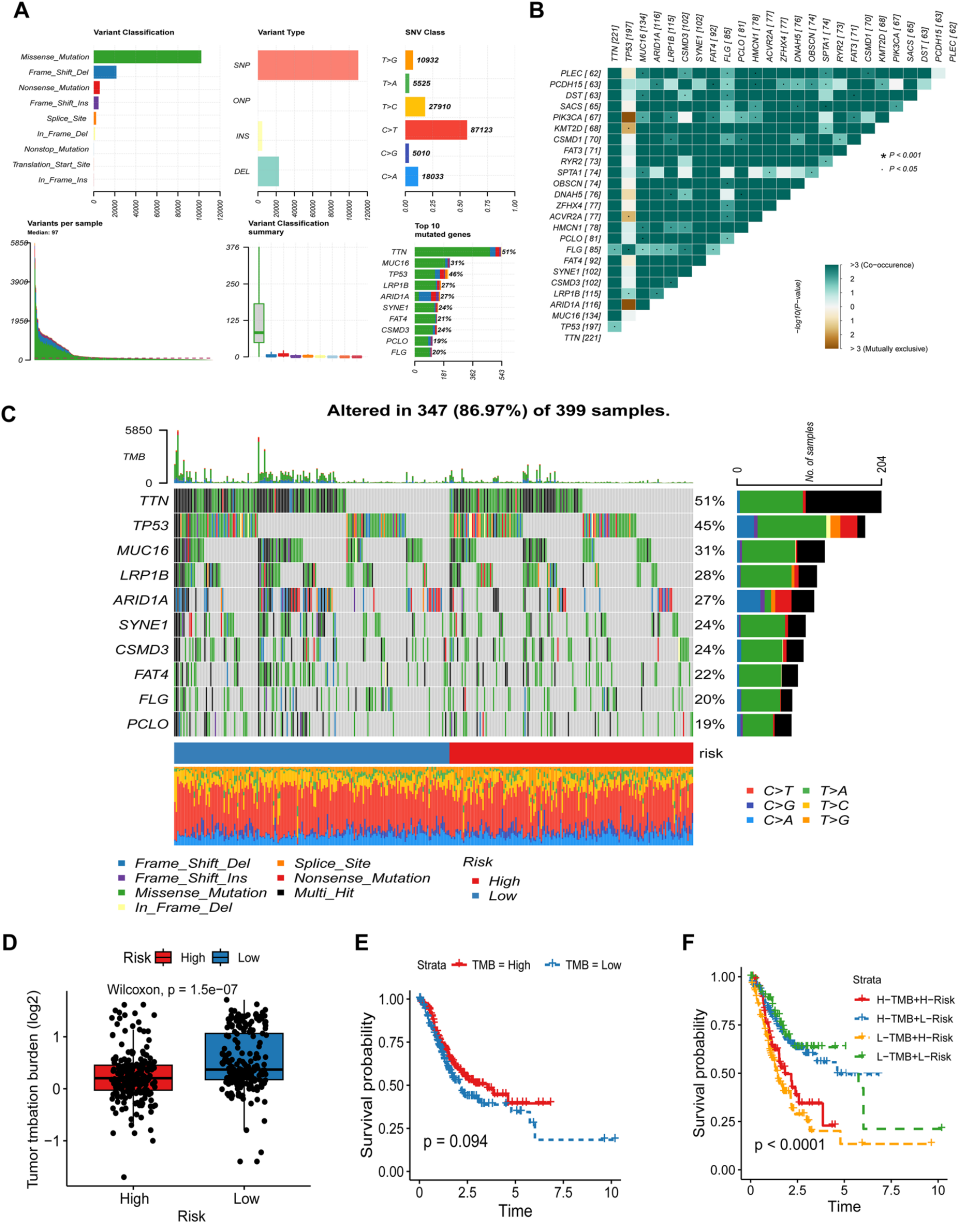
Somatic mutation characteristics, TMB survival analysis, and risk score. (**A**) General description of the mutation landscape in TCGA-STAD patients. (**B**) Genes with mutation effects that vary between low-risk and high-risk patient populations. (**C**) Top 10 genes' mutation landscapes in high- and low-risk populations. (**D**) TMB expression varies across populations at low and high-risk. (**E**) TMB group Kaplan–Meier curves for high- and low levels. (**F**) The four groups' Kaplan–Meier curves, broken down by risk score and TMB.

Figure 7A displays the whole profile of STAD mutations. The interaction of genetic mutations is shown in Fig. 7B, where the majority of genes show co-occurrence of mutations. TTN, TP53, and MUC16 were found to be the genes that were changed the most often in both subgroups of low-risk and high-risk patients when we additionally looked at genetic mutations (Fig. 7C). In the TCGA cohort, we investigated the connection between the tumor mutational burden (TMB) and TCMG-score. According to the findings, patients at high-risk had considerably lower TMB than patients at low-risk (Fig. 7D). There was no discernible difference in prognosis between the high-TMB and low-TMB groups according to the Kaplan–Meier analysis based on the median of the TMB values (*P* = 0.094) (Fig. 7E). However, the high TMB group exhibited a trend towards a better prognosis, and there are several plausible explanations for this observation. Firstly, elevated TMB levels may serve as an indicator of increased genetic mutation burden within tumor cells, which in turn could elicit stronger immune responses and enhanced anti-tumor effects. This heightened immune response may contribute to the suppression of tumor growth and metastasis, thereby improving patient outcomes. Secondly, the high TMB group might demonstrate a greater likelihood of responding to specific treatment modalities, such as immune checkpoint inhibitors. Consequently, the high TMB group may receive more efficacious therapies, resulting in a more favorable prognosis. he model's validity was demonstrated and the ideal prognostic groupings for clinical usage were found in the low-TMB and low-risk group (Fig. 7F). Patients were categorized into four groups, and a composite score that took the risk score and TMB into account was used to determine the patients' chances of survival.

### Construction of a nomogram for foreseeing survival

**Figure 8:**
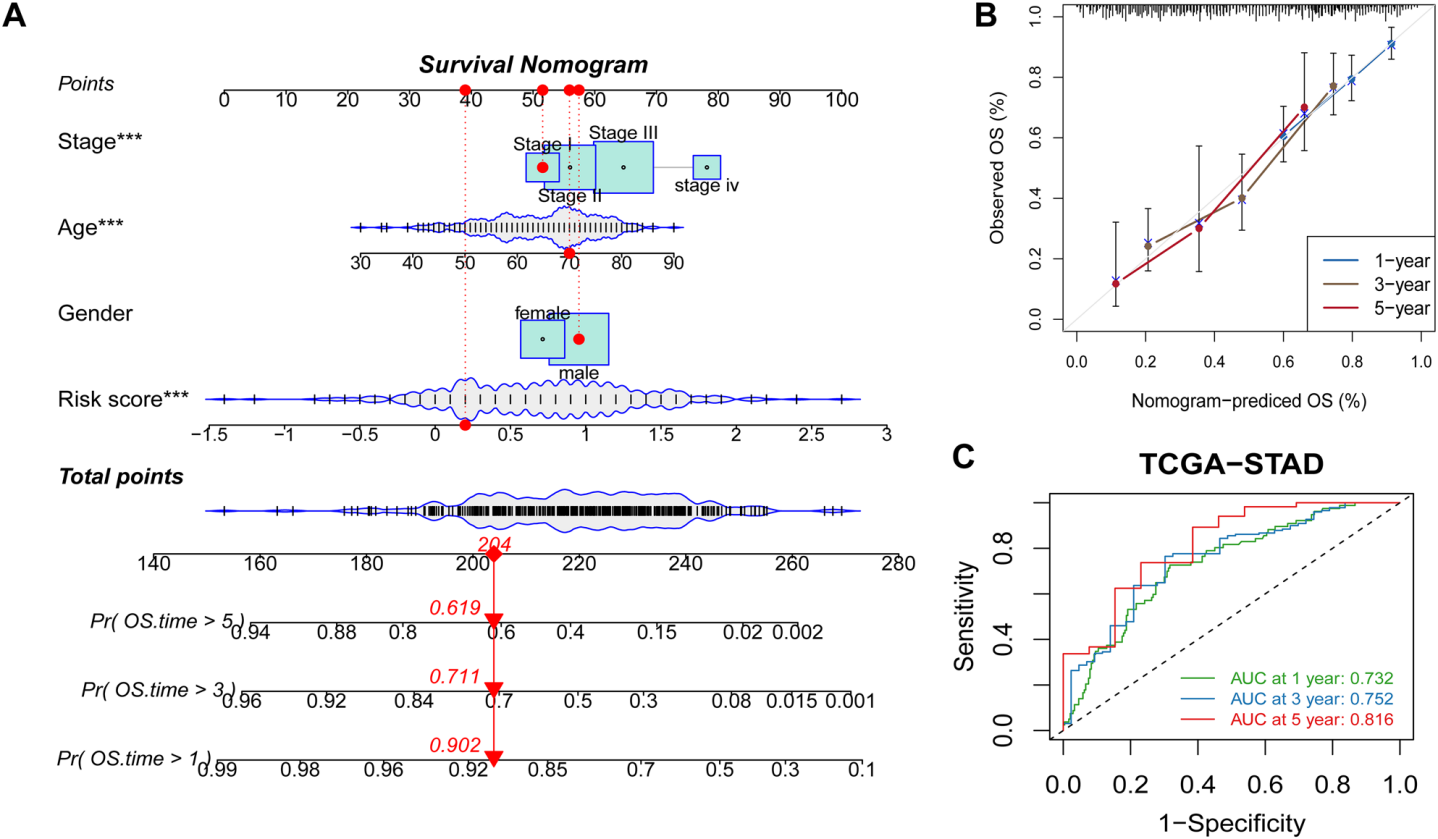
A nomogram's creation and validation. (**A**) Nomogram for predicting GC patients' 1, 3, and 5-year OS in the TCGA cohort. (**B**) The nomogram's calibration curves for forecasting 1, 3, and 5-year OS in the TCGA cohort. (**C**) ROC curves for projecting the TCGA cohorts' 1, 3, and 5-year ROC curves. ***P < 0.001.

To forecast the likelihood that GC patients will survive 1, 3, and 5 years from diagnosis, we combined clinical variables and TCMG-score (Fig. 8A). A 70-year-old male patient in stage I with a Risk score of 0.2, for instance, would have 204 points overall, which would indicate survival probability of 0.902, 0.711, and 0.619 at 1, 3, and 5 years, respectively. High concordance between actual and expected values was shown in calibration plots (Fig. 8B). Good accuracy for OS was found by AUC studies on the nomogram model for the TCGA cohorts, indicating that the TCMG-based nomogram may be an effective tool for predicting patient prognosis in clinical practice (Fig. 8C).

## Discussion

The prevalence of gastric cancer is rising in many nations, making it a frequent malignancy globally. Despite recent advances in GC management, its heterogeneous and aggressive characteristics hinder prognostic assessment. Finding novel biomarkers through screening is therefore essential and urgent in order to develop patient-specific medications and improve prognosis. Because it focuses on the levels of gene expression in specific cells rather than the average expression levels in bulk RNA-seq, the method of single-cell RNA sequencing has become effective for transcriptional stratification. In a range of cancers, including GC, it can identify cell subpopulations, specific biomarkers, and cell type heterogeneity. In this study, we thoroughly analyzed bulk RNA-seq and scream-seq data to develop a risk model for the efficacy of GC immunotherapy with good prognostic and predictive capabilities.

By examining various cell subpopulations, scRNA-seq technology has lately come to be acknowledged as a promising tool for examining tumor heterogeneity and discovering possible treatment targets. In the study, we used scRNA-seq analysis to investigate the T cell marker genes in GC and a training cohort to develop a prediction signature. Using different cohorts from the GEO dataset, we further verified the prediction capacity of this signature.

Immunotherapy is a powerful therapeutic approach for the management of cancer. The success of immune checkpoint inhibitors (ICIs) has raised interest in immunotherapy for gastric cancer. But it's still challenging to identify stomach cancer patients who might benefit from immunotherapy.

The study show that the high-risk group had a higher immunological score, estimated score, immune cell infiltration and somatic mutations compared to the low-risk group. In addition, the high-risk group had more immune-related pathways. Significantly, in the low-risk group, patients responded better to immunotherapy than patients in the high-risk group. These findings suggest that immune checkpoint blockade therapy might be more effective in treating low-risk patients.

The majority of the five T cell marker genes in the predictive signature in this study—MMP2, SERPINE1, CXCR4, CTLA4, and CXCL3—have been discovered to play a role in GC development and immune response. A zinc-dependent metalloproteinase called MMP2 has been connected to angiogenesis and cancer^16^. Higher levels of the protein MMP2 were shown to be associated with better survival in Li's cohort of patients with gastric cancer^17^. MMP2, also referred to as type IV collagenase, is released in the form of an inactive proenzyme that, when activated by hydrolysis, may break down gelatin and other proteins in the extracellular matrix, which is crucial for tumor invasion and metastasis^18^. Endothelial cells, monocytes, leukocytes, chondrocytes, platelets, osteoblasts, dermal fibroblasts, and keratinocytes are the main sources of MMP2 release^19^. As the main inhibitor of plasminogen activator, SERPINE1, also known as plasminogen activator inhibitor 1 (PAI-1), is essential for carcinogenesis^20^. SERPINE1 loss reduces tumor invasion and angiogenesis in animal models^21^. Higher levels of SERPINE1 in gastric cancer tissue relative to normal gastric tissue levels have been linked to a worse prognosis^22–25^. Both an invasive gastric cancer cell culture and gastric cancer tissues displayed significant levels of CXCR4 expression. In GC patients, higher levels of CXCR4 were linked to more advanced tumor stages and lower survival rates^26^. The immunosuppressive protein CTLA4 is expressed on the surface of T cells^27^. B7 is the CTLA4 ligand, and when it binds to the CTLA4 pathway, it inhibits and depletes T cells^28^. A reduction in immune function may occur from the CTLA4 pathway being overactivated^29^, followed by tumor cells evading the immune system. Anti-CTLA4 drugs can therefore stimulate T lymphocytes and kill tumor cells^30^. Vascular invasion and the development of tumor capsules are linked to CXCL3, also known as CINC-2 alpha^31^. It is strongly expressed during hepatic injury and inflammation, as well as in a number of tumor forms, includes hepatocellular carcinoma, severe breast cancer, colorectal cancer, prostate cancer, and melanoma^32–34^.

Five T cell marker genes were used to create the prognostic signature, which was verified in the TCGA-STAD and GEO cohorts. Consistent results were obtained in both populations, demonstrating the prognostic signature's resilience and reproducibility. Additionally, we created a nomogram that depicts and intuitively forecasts patients' chances of surviving for one, three, and five years. The nomogram's calibration plot showed greater prediction accuracy. As a result, this nomogram can make it easier to create customized examination plans for GC patients and to allocate medical resources as efficiently as possible.

We investigated the link between TCMG risk and TME in light of TME's crucial role in modulating anti-tumor responses and its significant influence on patient prognosis. In the high-risk group, our research showed a substantial decline in immunological scores and a concurrent increase in matrix scores. The high-risk group had elevated proportions of different immune cells, such as naive B cells, resting memory CD4 + T cells, monocytes, and resting mast cells, according to a further analysis of 22 immune cell infiltration levels. This finding suggests that these patients have a relatively active state of anti-tumor immunity. Additionally, immune checkpoint inhibitors have shown promise as lung cancer treatment targets. Our results demonstrated that TIDE expression was downregulated in low-risk individuals whereas PD-L1, PDCD-1, IDO1, LAG-3, ICOS, and TIGIT expression were elevated, demonstrating that those at low-risk may benefit more from immunotherapy. Collectively, our results show elevated immune responses and enhanced immune cell infiltration in high-risk individuals, highlighting their potential to benefit more from immunotherapy.

### Limitations

This study offers insightful information on the creation of fresh treatment plans for stomach cancer. However, several limitations should be acknowledged. Firstly, the analysis relied on retrospective cohort studies, and further validation in prospective cohorts is necessary to confirm the findings. Secondly, the investigation was limited to T cell marker genes, which may restrict the prognostic value of the signature due to the high spatial heterogeneity of the tumor immune microenvironment. Thirdly, the lack of functional data and immune-blocking therapy-treated GC datasets may introduce bias and preclude a comprehensive analysis of clinical and pathological parameters. To overcome these limitations, future studies should conduct prospective, double-blind, multi-center investigations on a broad scale to further corroborate the results.

## Conclusion

We have identified and validated a five-gene profile based on T cell markers that accurately predicts the prognosis and response to immunotherapy in GC patients. This signature can help identify suitable individuals who might benefit from immunotherapy and act as a predictive biomarker for individualized prediction in therapeutic decision-making. Our signature offers clinicians a robust tool to stratify patients based on their propensity to respond to immune therapy, which has the potential to improve patient outcomes and lessen unnecessary treatment-related harm.

### Supplementary Information

Supplementary Information.

## Data Availability

The entire set of findings presented here is based on information from TCGA, available at https://www.cancer.gov/tcga, and the GEO database, accessible at https://www.ncbi.nlm.nih.gov/geo/, with accession numbers GSE183904, GSE62254, GSE84437, and GSE84433.
