# Supplementary material for: Identification and validation of a T cell marker gene-based signature to predict prognosis and immunotherapy response in gastric cancer
Source: Sci Rep. 2023 Dec 4;13:21357. doi: 10.1038/s41598-023-48930-8 (PMC10696024; doi:10.1038/s41598-023-48930-8)
Supplement: Supplementary file 1 — Supplementary Information. [file 41598_2023_48930_MOESM1_ESM.pdf]

# Identification and Validation of a T Cell Marker Gene-Based Signature to Predict Prognosis and Immunotherapy Response in Gastric Cancer

Jinlin Zhong[1], Rongling Pan[1],Miao Gao[1], Yuqian Mo[1], Xin Peng[1], Guoxiao Liang[1],

Zixuan Chen[1], Jinlin Du[1]， Zhigang Huang*[1,2]

**Supplementary Table 1. 322 T-cell marker genes**

| gene | p_val | log2FC | pct.1 | pct.2 | p_val_adj |
|---|---|---|---|---|---|
| TNFRSF18 | 0 | 1.049575 | 0.228 | 0.142 | 0 |
| HSPG2 | 0 | -1.13126 | 0.014 | 0.18 | 0 |
| C1QA | 0 | -2.01963 | 0.033 | 0.148 | 0 |
| C1QC | 0 | -2.36517 | 0.037 | 0.15 | 0 |
| C1QB | 0 | -2.29661 | 0.036 | 0.152 | 0 |
| ID3 | 0 | -1.22736 | 0.154 | 0.415 | 0 |
| RUNX3 | 0 | 1.323282 | 0.429 | 0.13 | 0 |
| CD52 | 0 | 1.807242 | 0.739 | 0.263 | 0 |
| LCK | 0 | 1.699983 | 0.527 | 0.063 | 0 |
| CYR61 | 0 | -1.57253 | 0.012 | 0.216 | 0 |
| PTPN22 | 0 | 1.312587 | 0.385 | 0.092 | 0 |
| CD2 | 0 | 2.006463 | 0.578 | 0.069 | 0 |
| S100A11 | 0 | -1.32161 | 0.543 | 0.755 | 0 |
| S100A9 | 0 | -1.78901 | 0.025 | 0.148 | 0 |
| S100A8 | 0 | -2.13936 | 0.025 | 0.122 | 0 |
| S100A6 | 0 | -1.53702 | 0.791 | 0.813 | 0 |
| S100A4 | 0 | 1.060653 | 0.748 | 0.487 | 0 |
| S100A13 | 0 | -1.0731 | 0.028 | 0.301 | 0 |
| RAB13 | 0 | -1.01998 | 0.017 | 0.342 | 0 |
| RPS27 | 0 | 1.095893 | 0.995 | 0.974 | 0 |
| SH2D2A | 0 | 1.695173 | 0.481 | 0.072 | 0 |
| FCER1G | 0 | -1.59637 | 0.08 | 0.232 | 0 |
| CD247 | 0 | 1.624449 | 0.435 | 0.046 | 0 |
| XCL2 | 0 | 1.709226 | 0.169 | 0.016 | 0 |
| XCL1 | 0 | 1.751977 | 0.147 | 0.015 | 0 |
| CACYBP | 0 | 1.217515 | 0.615 | 0.505 | 0 |
| GLUL | 0 | -1.13733 | 0.19 | 0.417 | 0 |
| RGS1 | 0 | 1.10413 | 0.63 | 0.403 | 0 |
| PTPRC | 0 | 1.819697 | 0.804 | 0.343 | 0 |
| ELF3 | 0 | -1.54586 | 0.038 | 0.17 | 0 |
| G0S2 | 0 | -1.69076 | 0.028 | 0.187 | 0 |
| LINC01871 | 0 | 1.000674 | 0.211 | 0.018 | 0 |
| YPEL5 | 0 | 1.156335 | 0.679 | 0.542 | 0 |
| SRSF7 | 0 | 1.191637 | 0.762 | 0.638 | 0 |
| ZFP36L2 | 0 | 1.64507 | 0.878 | 0.738 | 0 |
| EFEMP1 | 0 | -1.06028 | 0.005 | 0.113 | 0 |
| GNLY | 0 | 2.984374 | 0.222 | 0.032 | 0 |
| CD8A | 0 | 2.156177 | 0.428 | 0.046 | 0 |
| CD8B | 0 | 1.354343 | 0.278 | 0.03 | 0 |
| IGKC | 0 | -3.95187 | 0.246 | 0.395 | 0 |
| DUSP2 | 0 | 1.654692 | 0.785 | 0.459 | 0 |
| ZAP70 | 0 | 1.195594 | 0.343 | 0.038 | 0 |
| PDCL3 | 0 | 1.135251 | 0.394 | 0.218 | 0 |
| IL1B | 0 | -2.78945 | 0.036 | 0.19 | 0 |
| IL1RN | 0 | -1.19915 | 0.01 | 0.115 | 0 |
| CXCR4 | 0 | 2.164958 | 0.796 | 0.318 | 0 |
| CYTIP | 0 | 1.234258 | 0.626 | 0.367 | 0 |
| WIPF1 | 0 | 1.104385 | 0.501 | 0.294 | 0 |
| COL3A1 | 0 | -2.72739 | 0.05 | 0.202 | 0 |
| STAT4 | 0 | 1.08786 | 0.335 | 0.08 | 0 |
| STK17B | 0 | 1.435877 | 0.634 | 0.345 | 0 |
| CTLA4 | 0 | 1.0141 | 0.193 | 0.024 | 0 |
| ICOS | 0 | 1.199326 | 0.247 | 0.029 | 0 |
| FN1 | 0 | -1.8707 | 0.017 | 0.198 | 0 |
| IGFBP2 | 0 | -1.1477 | 0.027 | 0.194 | 0 |
| IGFBP5 | 0 | -1.74102 | 0.012 | 0.119 | 0 |

| | | | | | |
|---|---|---|---|---|---|
| TUBA4A | 0 | 1.688903 | 0.564 | 0.31 | 0 |
| CXCR6 | 0 | 1.153407 | 0.235 | 0.023 | 0 |
| CBLB | 0 | 1.033874 | 0.336 | 0.136 | 0 |
| CD96 | 0 | 1.317518 | 0.372 | 0.041 | 0 |
| CCDC80 | 0 | -1.36784 | 0.009 | 0.127 | 0 |
| TIGIT | 0 | 1.299954 | 0.273 | 0.032 | 0 |
| TM4SF1 | 0 | -1.62099 | 0.021 | 0.252 | 0 |
| MBNL1 | 0 | 1.016487 | 0.563 | 0.427 | 0 |
| PPP1R2 | 0 | 1.149805 | 0.534 | 0.414 | 0 |
| S100P | 0 | -1.71504 | 0.075 | 0.179 | 0 |
| RHOH | 0 | 1.063563 | 0.5 | 0.268 | 0 |
| HOPX | 0 | 1.040142 | 0.255 | 0.073 | 0 |
| IGFBP7 | 0 | -3.40522 | 0.089 | 0.299 | 0 |
| JCHAIN | 0 | -3.7193 | 0.17 | 0.406 | 0 |
| CXCL8 | 0 | -3.05429 | 0.054 | 0.264 | 0 |
| CXCL1 | 0 | -1.26731 | 0.019 | 0.13 | 0 |
| CXCL3 | 0 | -2.68258 | 0.038 | 0.246 | 0 |
| CXCL2 | 0 | -2.58924 | 0.04 | 0.277 | 0 |
| G3BP2 | 0 | 1.280846 | 0.537 | 0.363 | 0 |
| CNOT6L | 0 | 1.279435 | 0.423 | 0.178 | 0 |
| SPARCL1 | 0 | -1.87959 | 0.018 | 0.193 | 0 |
| SPP1 | 0 | -3.33852 | 0.023 | 0.11 | 0 |
| IL7R | 0 | 2.20536 | 0.504 | 0.131 | 0 |
| FYB1 | 0 | 1.418157 | 0.573 | 0.18 | 0 |
| PTGER4 | 0 | 1.067065 | 0.348 | 0.143 | 0 |
| SELENOP | 0 | -1.38958 | 0.023 | 0.247 | 0 |
| EMB | 0 | 1.040872 | 0.364 | 0.146 | 0 |
| PARP8 | 0 | 1.102901 | 0.346 | 0.117 | 0 |
| GZMK | 0 | 1.802665 | 0.291 | 0.036 | 0 |
| GZMA | 0 | 2.827013 | 0.524 | 0.055 | 0 |
| PDE4D | 0 | 1.091293 | 0.358 | 0.142 | 0 |
| PIK3R1 | 0 | 1.367462 | 0.418 | 0.209 | 0 |
| VCAN | 0 | -1.24147 | 0.009 | 0.182 | 0 |
| TGFBI | 0 | -1.02976 | 0.01 | 0.2 | 0 |
| EGR1 | 0 | -1.0087 | 0.278 | 0.58 | 0 |
| MZB1 | 0 | -1.86494 | 0.081 | 0.287 | 0 |
| CYSTM1 | 0 | -1.36104 | 0.153 | 0.439 | 0 |
| CD14 | 0 | -1.35274 | 0.008 | 0.156 | 0 |
| NR3C1 | 0 | 1.068068 | 0.462 | 0.336 | 0 |
| SPINK1 | 0 | -1.6236 | 0.058 | 0.145 | 0 |
| SPARC | 0 | -2.96517 | 0.06 | 0.277 | 0 |
| ITK | 0 | 1.042689 | 0.283 | 0.039 | 0 |
| HLA-A | 0 | 1.133087 | 0.975 | 0.897 | 0 |
| IER3 | 0 | -1.79563 | 0.153 | 0.53 | 0 |
| HLA-B | 0 | 1.069317 | 0.982 | 0.926 | 0 |
| LTB | 0 | 1.42981 | 0.357 | 0.107 | 0 |
| AIF1 | 0 | -1.48421 | 0.037 | 0.192 | 0 |
| HLA-DRA | 0 | -2.42705 | 0.283 | 0.442 | 0 |
| HLA-DQA1 | 0 | -1.1357 | 0.196 | 0.33 | 0 |
| HLA-DQB1 | 0 | -1.05246 | 0.137 | 0.286 | 0 |
| CD24 | 0 | -1.03165 | 0.036 | 0.169 | 0 |
| FYN | 0 | 1.615582 | 0.523 | 0.131 | 0 |
| MARCKS | 0 | -1.06967 | 0.021 | 0.384 | 0 |
| CTGF | 0 | -1.59664 | 0.012 | 0.203 | 0 |
| SGK1 | 0 | -1.20555 | 0.099 | 0.393 | 0 |
| TNFAIP3 | 0 | 1.624405 | 0.809 | 0.513 | 0 |
| SYTL3 | 0 | 1.544981 | 0.468 | 0.116 | 0 |
| TAGAP | 0 | 1.200982 | 0.474 | 0.263 | 0 |

| | | | | | |
|---|---|---|---|---|---|
| SOD2 | 0 | -1.56835 | 0.304 | 0.535 | 0 |
| AGR2 | 0 | -2.04454 | 0.124 | 0.233 | 0 |
| STK17A | 0 | 1.344867 | 0.578 | 0.296 | 0 |
| IGFBP3 | 0 | -1.51899 | 0.051 | 0.147 | 0 |
| CLDN4 | 0 | -1.35073 | 0.032 | 0.126 | 0 |
| GNG11 | 0 | -1.0982 | 0.009 | 0.177 | 0 |
| COL1A2 | 0 | -2.68571 | 0.046 | 0.199 | 0 |
| SERPINE1 | 0 | -1.1974 | 0.017 | 0.159 | 0 |
| CAV1 | 0 | -1.26274 | 0.012 | 0.23 | 0 |
| AC016831.7 | 0 | 1.269376 | 0.5 | 0.254 | 0 |
| CALD1 | 0 | -2.01757 | 0.026 | 0.237 | 0 |
| TRBC1 | 0 | 1.613151 | 0.361 | 0.045 | 0 |
| TRBC2 | 0 | 1.812718 | 0.526 | 0.09 | 0 |
| RARRES2 | 0 | -1.95126 | 0.009 | 0.169 | 0 |
| TMEM176B | 0 | -1.65078 | 0.012 | 0.295 | 0 |
| TMEM176A | 0 | -1.57828 | 0.012 | 0.283 | 0 |
| PRDX4 | 0 | -1.14235 | 0.084 | 0.516 | 0 |
| TIMP1 | 0 | -3.75607 | 0.176 | 0.652 | 0 |
| ITM2A | 0 | 1.106389 | 0.391 | 0.181 | 0 |
| TSC22D3 | 0 | 1.159186 | 0.795 | 0.657 | 0 |
| BGN | 0 | -1.71707 | 0.021 | 0.168 | 0 |
| SSR4 | 0 | -1.37672 | 0.617 | 0.816 | 0 |
| CTSB | 0 | -2.10961 | 0.167 | 0.526 | 0 |
| DOK2 | 0 | 1.246003 | 0.393 | 0.122 | 0 |
| CLU | 0 | -1.22856 | 0.037 | 0.198 | 0 |
| DUSP4 | 0 | 1.758261 | 0.446 | 0.211 | 0 |
| SARAF | 0 | 1.647161 | 0.908 | 0.75 | 0 |
| LEPROTL1 | 0 | 1.562024 | 0.616 | 0.319 | 0 |
| PLAT | 0 | -1.19721 | 0.01 | 0.14 | 0 |
| SLA | 0 | 1.115666 | 0.336 | 0.112 | 0 |
| DNAJA1 | 0 | 1.186825 | 0.844 | 0.774 | 0 |
| TPM2 | 0 | -1.4037 | 0.022 | 0.217 | 0 |
| ANXA1 | 0 | 1.174684 | 0.564 | 0.465 | 0 |
| CTSL | 0 | -1.73878 | 0.037 | 0.308 | 0 |
| KLF4 | 0 | -1.30473 | 0.036 | 0.341 | 0 |
| GSN | 0 | -1.7637 | 0.039 | 0.4 | 0 |
| IFITM1 | 0 | 1.060733 | 0.79 | 0.485 | 0 |
| IFITM3 | 0 | -2.68261 | 0.205 | 0.555 | 0 |
| CD151 | 0 | -1.12511 | 0.101 | 0.43 | 0 |
| PHLDA2 | 0 | -1.0496 | 0.083 | 0.32 | 0 |
| MDK | 0 | -1.42005 | 0.038 | 0.284 | 0 |
| SERPING1 | 0 | -1.42582 | 0.01 | 0.243 | 0 |
| CD6 | 0 | 1.254176 | 0.346 | 0.041 | 0 |
| RARRES3 | 0 | 1.302777 | 0.571 | 0.337 | 0 |
| NEAT1 | 0 | -1.09638 | 0.601 | 0.808 | 0 |
| CTSW | 0 | 1.163836 | 0.251 | 0.046 | 0 |
| PTPRCAP | 0 | 1.928874 | 0.758 | 0.291 | 0 |
| GSTP1 | 0 | -1.10666 | 0.475 | 0.794 | 0 |
| PRSS23 | 0 | -1.17074 | 0.022 | 0.196 | 0 |
| NNMT | 0 | -1.20898 | 0.01 | 0.206 | 0 |
| TAGLN | 0 | -1.9259 | 0.03 | 0.171 | 0 |
| CD3E | 0 | 2.636054 | 0.813 | 0.1 | 0 |
| CD3D | 0 | 2.382393 | 0.689 | 0.082 | 0 |
| CD3G | 0 | 1.508843 | 0.428 | 0.045 | 0 |
| THY1 | 0 | -1.11054 | 0.01 | 0.153 | 0 |
| HSPA8 | 0 | 1.241454 | 0.964 | 0.9 | 0 |
| ETS1 | 0 | 1.07553 | 0.399 | 0.17 | 0 |
| CREM | 0 | 1.857344 | 0.757 | 0.408 | 0 |

| | | | | | |
|---|---|---|---|---|---|
| PRF1 | 0 | 1.69527 | 0.351 | 0.036 | 0 |
| PSAP | 0 | -1.73664 | 0.322 | 0.73 | 0 |
| SPOCK2 | 0 | 1.726639 | 0.501 | 0.09 | 0 |
| ADIRF | 0 | -1.05194 | 0.016 | 0.189 | 0 |
| PDLIM1 | 0 | -1.26964 | 0.044 | 0.364 | 0 |
| PDCD4 | 0 | 1.214728 | 0.536 | 0.359 | 0 |
| CD9 | 0 | -1.32638 | 0.078 | 0.385 | 0 |
| LAG3 | 0 | 1.34948 | 0.288 | 0.061 | 0 |
| C1S | 0 | -1.38112 | 0.007 | 0.168 | 0 |
| A2M | 0 | -1.67903 | 0.014 | 0.238 | 0 |
| KLRB1 | 0 | 2.050533 | 0.373 | 0.043 | 0 |
| CLEC2D | 0 | 1.03041 | 0.308 | 0.068 | 0 |
| CD69 | 0 | 1.461721 | 0.683 | 0.272 | 0 |
| CLEC2B | 0 | 1.34758 | 0.527 | 0.316 | 0 |
| KLRD1 | 0 | 1.439882 | 0.261 | 0.025 | 0 |
| MGP | 0 | -2.39526 | 0.039 | 0.194 | 0 |
| ARHGDIB | 0 | 1.500328 | 0.847 | 0.528 | 0 |
| KRT8 | 0 | -2.05796 | 0.118 | 0.33 | 0 |
| KRT18 | 0 | -1.96361 | 0.1 | 0.335 | 0 |
| CD63 | 0 | -1.38865 | 0.329 | 0.733 | 0 |
| ARHGAP9 | 0 | 1.176256 | 0.382 | 0.11 | 0 |
| IFNG | 0 | 1.876021 | 0.214 | 0.022 | 0 |
| LYZ | 0 | -2.7881 | 0.111 | 0.35 | 0 |
| TSPAN8 | 0 | -1.03652 | 0.034 | 0.146 | 0 |
| LUM | 0 | -2.25604 | 0.014 | 0.134 | 0 |
| DCN | 0 | -2.21802 | 0.017 | 0.165 | 0 |
| BTG1 | 0 | 1.705278 | 0.964 | 0.799 | 0 |
| HSP90B1 | 0 | -1.13234 | 0.473 | 0.784 | 0 |
| POSTN | 0 | -2.02121 | 0.007 | 0.112 | 0 |
| ELF1 | 0 | 1.225516 | 0.627 | 0.449 | 0 |
| RGCC | 0 | 1.482993 | 0.64 | 0.398 | 0 |
| LCP1 | 0 | 1.149302 | 0.575 | 0.25 | 0 |
| COL4A1 | 0 | -1.3358 | 0.018 | 0.156 | 0 |
| COL4A2 | 0 | -1.31511 | 0.012 | 0.181 | 0 |
| RNASE1 | 0 | -1.74172 | 0.032 | 0.213 | 0 |
| GZMH | 0 | 1.726238 | 0.31 | 0.032 | 0 |
| GZMB | 0 | 2.378687 | 0.406 | 0.046 | 0 |
| FAM177A1 | 0 | 1.019656 | 0.484 | 0.41 | 0 |
| RPS29 | 0 | 1.050539 | 0.94 | 0.884 | 0 |
| GNG2 | 0 | 1.480823 | 0.534 | 0.202 | 0 |
| LGALS3 | 0 | -1.0049 | 0.363 | 0.608 | 0 |
| PRKCH | 0 | 1.012282 | 0.323 | 0.082 | 0 |
| NPC2 | 0 | -1.83734 | 0.151 | 0.647 | 0 |
| BATF | 0 | 1.409395 | 0.303 | 0.11 | 0 |
| GPR65 | 0 | 1.115459 | 0.368 | 0.131 | 0 |
| LGMN | 0 | -1.0505 | 0.029 | 0.319 | 0 |
| IFI27 | 0 | -2.21616 | 0.099 | 0.388 | 0 |
| SERPINA1 | 0 | -1.53293 | 0.027 | 0.208 | 0 |
| EVL | 0 | 1.691506 | 0.592 | 0.186 | 0 |
| PPP2R5C | 0 | 1.062745 | 0.408 | 0.244 | 0 |
| CRIP2 | 0 | -1.22391 | 0.028 | 0.232 | 0 |
| IGHA2 | 0 | -3.61901 | 0.099 | 0.263 | 0 |
| IGHA1 | 0 | -3.86454 | 0.27 | 0.442 | 0 |
| C15orf48 | 0 | -1.73316 | 0.034 | 0.223 | 0 |
| ANXA2 | 0 | -1.2124 | 0.295 | 0.593 | 0 |
| RORA | 0 | 1.211627 | 0.363 | 0.136 | 0 |
| TPM1 | 0 | -1.39002 | 0.055 | 0.318 | 0 |
| BCL2A1 | 0 | -1.41953 | 0.115 | 0.227 | 0 |

| | | | | | |
|---|---|---|---|---|---|
| IL32 | 0 | 2.291676 | 0.88 | 0.367 | 0 |
| SOCS1 | 0 | 1.075391 | 0.458 | 0.272 | 0 |
| NUPR1 | 0 | -1.02081 | 0.012 | 0.204 | 0 |
| LAT | 0 | 1.089483 | 0.359 | 0.088 | 0 |
| CORO1A | 0 | 1.772127 | 0.769 | 0.339 | 0 |
| 1-Sep | 0 | 1.360113 | 0.498 | 0.178 | 0 |
| MMP2 | 0 | -1.12231 | 0.004 | 0.152 | 0 |
| MT1G | 0 | -1.19754 | 0.031 | 0.124 | 0 |
| SLC7A5 | 0 | 1.021622 | 0.427 | 0.277 | 0 |
| ACAP1 | 0 | 1.168906 | 0.42 | 0.149 | 0 |
| CD68 | 0 | -1.55611 | 0.028 | 0.273 | 0 |
| MFAP4 | 0 | -1.3048 | 0.003 | 0.111 | 0 |
| CCL2 | 0 | -1.71557 | 0.007 | 0.163 | 0 |
| CCL5 | 0 | 3.279779 | 0.717 | 0.152 | 0 |
| CCL4 | 0 | 1.015692 | 0.483 | 0.244 | 0 |
| IGFBP4 | 0 | -1.82236 | 0.049 | 0.27 | 0 |
| KRT19 | 0 | -1.87134 | 0.088 | 0.267 | 0 |
| RAMP2 | 0 | -1.02381 | 0.008 | 0.128 | 0 |
| GRN | 0 | -1.39534 | 0.069 | 0.491 | 0 |
| SKAP1 | 0 | 1.077976 | 0.341 | 0.09 | 0 |
| COL1A1 | 0 | -3.07987 | 0.055 | 0.21 | 0 |
| LIMD2 | 0 | 1.137016 | 0.523 | 0.287 | 0 |
| LGALS3BP | 0 | -1.09404 | 0.047 | 0.311 | 0 |
| CD7 | 0 | 2.805947 | 0.744 | 0.132 | 0 |
| LDLRAD4 | 0 | 1.206753 | 0.354 | 0.149 | 0 |
| RNF125 | 0 | 1.005767 | 0.283 | 0.067 | 0 |
| TCF4 | 0 | -1.03465 | 0.019 | 0.328 | 0 |
| CST3 | 0 | -3.29574 | 0.126 | 0.632 | 0 |
| CST7 | 0 | 2.285314 | 0.617 | 0.107 | 0 |
| ID1 | 0 | -1.20885 | 0.082 | 0.33 | 0 |
| MYL9 | 0 | -1.68305 | 0.025 | 0.24 | 0 |
| SLA2 | 0 | 1.21625 | 0.289 | 0.032 | 0 |
| MAFB | 0 | -1.142 | 0.013 | 0.217 | 0 |
| STK4 | 0 | 1.044995 | 0.505 | 0.327 | 0 |
| SLPI | 0 | -1.20566 | 0.012 | 0.116 | 0 |
| PLTP | 0 | -1.37953 | 0.011 | 0.23 | 0 |
| GZMM | 0 | 1.34917 | 0.361 | 0.04 | 0 |
| CFD | 0 | -1.72011 | 0.019 | 0.202 | 0 |
| C3 | 0 | -1.00025 | 0.009 | 0.116 | 0 |
| ADGRE5 | 0 | 1.188873 | 0.488 | 0.298 | 0 |
| HCST | 0 | 1.647268 | 0.657 | 0.231 | 0 |
| TYROBP | 0 | -1.45434 | 0.146 | 0.273 | 0 |
| SPINT2 | 0 | -1.05374 | 0.06 | 0.322 | 0 |
| LGALS4 | 0 | -1.07736 | 0.038 | 0.146 | 0 |
| CD79A | 0 | -1.29514 | 0.043 | 0.295 | 0 |
| ARHGEF1 | 0 | 1.100244 | 0.466 | 0.257 | 0 |
| PLAUR | 0 | -1.94953 | 0.062 | 0.361 | 0 |
| APOE | 0 | -2.90662 | 0.063 | 0.257 | 0 |
| APOC1 | 0 | -1.93193 | 0.04 | 0.21 | 0 |
| FTL | 0 | -2.09316 | 0.939 | 0.973 | 0 |
| FCGRT | 0 | -1.26982 | 0.05 | 0.454 | 0 |
| NKG7 | 0 | 2.877995 | 0.59 | 0.091 | 0 |
| ZNF331 | 0 | 1.022427 | 0.546 | 0.317 | 0 |
| IGLV6-57 | 0 | -2.94928 | 0.038 | 0.118 | 0 |
| IGLV3-1 | 0 | -3.00599 | 0.036 | 0.119 | 0 |
| IGLL5 | 0 | -1.78695 | 0.021 | 0.191 | 0 |
| IGLC2 | 0 | -3.81195 | 0.104 | 0.215 | 0 |
| IGLC3 | 0 | -3.27185 | 0.054 | 0.146 | 0 |

| | | | | | |
|---|---|---|---|---|---|
| DERL3 | 0 | -1.37541 | 0.026 | 0.267 | 0 |
| TIMP3 | 0 | -1.01219 | 0.006 | 0.168 | 0 |
| IL2RB | 0 | 1.262904 | 0.329 | 0.039 | 0 |
| RAC2 | 0 | 1.496962 | 0.74 | 0.429 | 0 |
| LGALS1 | 0 | -1.62417 | 0.399 | 0.709 | 0 |
| APOBEC3G | 0 | 1.032054 | 0.305 | 0.126 | 0 |
| SAMSN1 | 0 | 1.170239 | 0.569 | 0.28 | 0 |
| APP | 0 | -1.06885 | 0.04 | 0.337 | 0 |
| SOD1 | 0 | 1.069931 | 0.745 | 0.651 | 0 |
| COL6A1 | 0 | -1.21119 | 0.009 | 0.19 | 0 |
| COL6A2 | 0 | -1.45979 | 0.019 | 0.203 | 0 |
| IGHM | 3.97E-270 | -2.8082 | 0.06 | 0.13 | 9.80E-266 |
| TFF2 | 7.65E-265 | -2.36693 | 0.078 | 0.153 | 1.89E-260 |
| TFF1 | 1.93E-263 | -2.79818 | 0.169 | 0.27 | 4.75E-259 |
| ACTA2 | 1.72E-262 | -1.53334 | 0.078 | 0.151 | 4.23E-258 |
| PHGR1 | 4.13E-230 | -1.08475 | 0.067 | 0.134 | 1.02E-225 |
| IGKV3-11 | 1.25E-186 | -3.16552 | 0.061 | 0.119 | 3.08E-182 |
| HLA-DRB1 | 1.08E-164 | -1.28311 | 0.415 | 0.469 | 2.67E-160 |
| MUC5AC | 3.79E-160 | -1.9928 | 0.16 | 0.238 | 9.35E-156 |
| HLA-DPA1 | 1.06E-155 | -1.2338 | 0.386 | 0.462 | 2.61E-151 |
| TNFRSF4 | 2.81E-139 | 1.036704 | 0.168 | 0.124 | 6.92E-135 |
| IGKV1-5 | 7.71E-136 | -3.08282 | 0.103 | 0.163 | 1.90E-131 |
| CD74 | 1.17E-130 | -1.42218 | 0.804 | 0.817 | 2.88E-126 |
| IGKV3-15 | 1.06E-128 | -3.40868 | 0.055 | 0.1 | 2.61E-124 |
| IGKV4-1 | 1.00E-119 | -3.39823 | 0.114 | 0.174 | 2.47E-115 |
| CCL3 | 5.81E-119 | -1.55721 | 0.144 | 0.197 | 1.43E-114 |
| IGLV2-14 | 2.78E-110 | -3.17218 | 0.08 | 0.129 | 6.86E-106 |
| IGHG1 | 1.95E-100 | -3.1225 | 0.139 | 0.194 | 4.80E-96 |
| PGC | 3.92E-86 | -3.15031 | 0.136 | 0.187 | 9.65E-82 |
| GKN1 | 6.90E-72 | -1.88194 | 0.077 | 0.113 | 1.70E-67 |
| IGLV1-40 | 8.42E-66 | -2.86957 | 0.072 | 0.108 | 2.08E-61 |
| IGKV3-20 | 2.40E-59 | -3.70269 | 0.178 | 0.228 | 5.92E-55 |
| HLA-DPB1 | 3.15E-50 | -1.079 | 0.383 | 0.408 | 7.78E-4 |
| CCL3L1 | 9.99E-50 | -1.16219 | 0.08 | 0.108 | 2.46E-4 |

| gene | HazardRatio(95%CI) | z | pvalue |
|---|---|---|---|
| FYN | 1.28097 (1.009-1.009) | 2.037078 | 0.042 |
| DCN | 1.17571 (1.045-1.045) | 2.685783 | 0.0072 |
| VCAN | 1.26554 (1.102-1.102) | 3.329166 | 0.00087 |
| MMP2 | 1.12538 (1.002-1.002) | 1.99079 | 0.047 |
| RGS1 | 1.2133 (1.063-1.063) | 2.867478 | 0.0041 |
| CCDC80 | 1.15165 (1.047-1.047) | 2.893936 | 0.0038 |
| CREM | 1.53326 (1.082-1.082) | 2.40481 | 0.016 |
| LGALS1 | 1.18132 (1.019-1.019) | 2.213459 | 0.027 |
| TIMP3 | 1.15644 (1.025-1.025) | 2.358024 | 0.018 |
| PLTP | 1.23478 (1.081-1.081) | 3.110951 | 0.0019 |
| MYL9 | 1.11978 (1.033-1.033) | 2.758782 | 0.0058 |
| TIMP1 | 1.17228 (1.009-1.009) | 2.080263 | 0.038 |
| PLAT | 1.25857 (1.027-1.027) | 2.21701 | 0.027 |
| CAV1 | 1.195 (1.066-1.066) | 3.062474 | 0.0022 |
| SERPINE1 | 1.25753 (1.134-1.134) | 4.333466 | 1.50E-05 |
| ACTA2 | 1.13894 (1.038-1.038) | 2.748731 | 0.006 |
| COL1A1 | 1.13371 (1.019-1.019) | 2.306327 | 0.021 |
| CD69 | 1.21061 (1.015-1.015) | 2.130737 | 0.033 |
| MGP | 1.13642 (1.044-1.044) | 2.940725 | 0.0033 |
| SPARC | 1.26671 (1.098-1.098) | 3.248566 | 0.0012 |
| NR3C1 | 1.35375 (1.098-1.098) | 2.83133 | 0.0046 |
| EFEMP1 | 1.2163 (1.097-1.097) | 3.713847 | 2.00E-04 |
| FN1 | 1.158 (1.045-1.045) | 2.795047 | 0.0052 |
| IGFBP5 | 1.18697 (1.056-1.056) | 2.867201 | 0.0041 |
| NPC2 | 1.39096 (1.073-1.073) | 2.491418 | 0.013 |
| YPEL5 | 1.44093 (1.01-1.01) | 2.012045 | 0.044 |
| CXCR4 | 1.23165 (1.08-1.08) | 3.110027 | 0.0019 |
| CALD1 | 1.16432 (1.052-1.052) | 2.937756 | 0.0033 |
| ADGRE5 | 0.736722 (0.583-0.583) | -2.55018 | 0.011 |
| ID1 | 0.84218 (0.74-0.74) | -2.59994 | 0.0093 |
| GNG11 | 1.38118 (1.148-1.148) | 3.429817 | 6.00E-04 |
| RNASE1 | 1.19922 (1.061-1.061) | 2.913153 | 0.0036 |
| ZNF331 | 1.37618 (1.118-1.118) | 3.006023 | 0.0026 |
| RAMP2 | 1.25304 (1.06-1.06) | 2.635204 | 0.0084 |
| POSTN | 1.19063 (1.057-1.057) | 2.877449 | 0.004 |
| COL4A2 | 1.23099 (1.04-1.04) | 2.421505 | 0.015 |
| LUM | 1.19955 (1.061-1.061) | 2.898953 | 0.0037 |
| IGFBP4 | 1.14469 (1.013-1.013) | 2.159872 | 0.031 |
| COL6A2 | 1.17828 (1.031-1.031) | 2.400619 | 0.016 |
| TAGLN | 1.1127 (1.026-1.026) | 2.571538 | 0.01 |
| PRSS23 | 1.26976 (1.082-1.082) | 2.918892 | 0.0035 |
| SPARCL1 | 1.15862 (1.055-1.055) | 3.06671 | 0.0022 |
| THY1 | 1.20331 (1.036-1.036) | 2.416877 | 0.016 |
| TSC22D3 | 1.19625 (1.038-1.038) | 2.468505 | 0.014 |
| IGFBP7 | 1.31228 (1.122-1.122) | 3.402265 | 0.00067 |
| CTLA4 | 0.797555 (0.648-0.648) | -2.13774 | 0.033 |
| CXCL3 | 0.87946 (0.798-0.798) | -2.58952 | 0.0096 |
| COL1A2 | 1.16278 (1.035-1.035) | 2.534728 | 0.011 |
| MFAP4 | 1.09996 (1.008-1.008) | 2.132006 | 0.033 |
| NNMT | 1.22296 (1.077-1.077) | 3.114468 | 0.0018 |
| COL3A1 | 1.17086 (1.046-1.046) | 2.743914 | 0.0061 |
| HOPX | 1.15516 (1.001-1.001) | 1.978166 | 0.048 |
| A2M | 1.1987 (1.047-1.047) | 2.629609 | 0.0085 |
| C1S | 1.15184 (1.005-1.005) | 2.037946 | 0.042 |
| BGN | 1.18686 (1.049-1.049) | 2.72724 | 0.0064 |
| TNFRSF18 | 0.822892 (0.684-0.684) | -2.07368 | 0.038 |
| COL4A1 | 1.26841 (1.061-1.061) | 2.616499 | 0.0089 |
| TCF4 | 1.36013 (1.066-1.066) | 2.474712 | 0.013 |
| TPM2 | 1.1091 (1.013-1.013) | 2.236751 | 0.025 |
| SELENOP | 1.15034 (1.005-1.005) | 2.03062 | 0.042 |
| MARCKS | 1.65608 (1.244-1.244) | 3.459539 | 0.00054 |

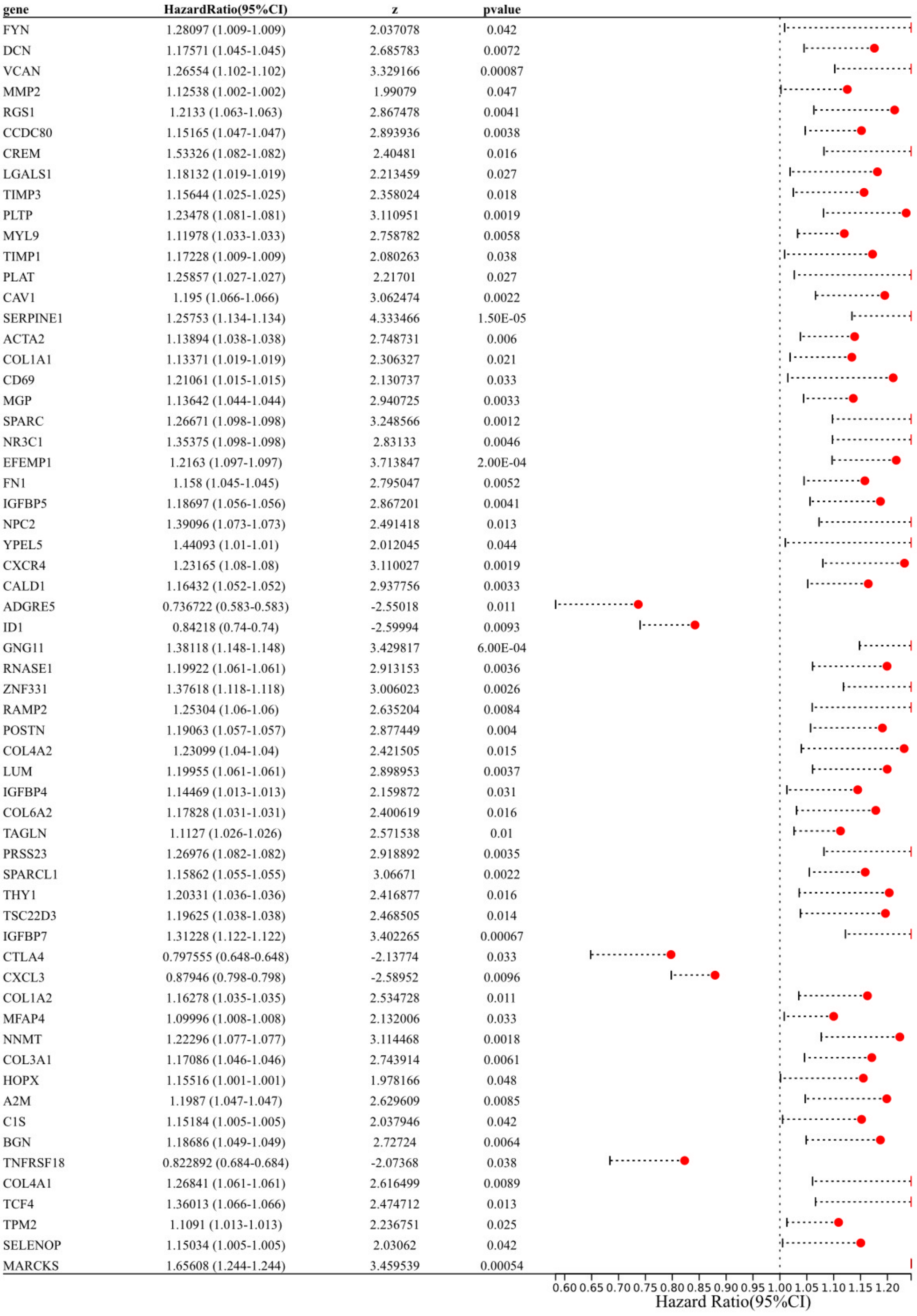


**Supplementary Figure 1**. 61 genes associated with overall survival.
